# The Cult of the Ugly

**Published:** 1919-03-15

**Authors:** 


### THE CULT OF THE UGLY.

### Let Parents and Teachers Beware.

A certain professor recently in a lecture asserted that certain forms of modern art were insanity or manifestations of insanity and infectious in character. This may or may not be so. In the newspaper report only the assertion was given and not the arguments. There is, however, a certain phenomenon of a curious nature that the assertion naturally calls to mind. It is the admiration for the ugly and unnatural, especially in children's toys and in some of the illustrations of some of the weekly papers. So far as the illustrations are concerned no doubt a reason for their production is that to an artist of some facility it is easy to draw a quite unnatural young woman and companions for her, though his skill be far below that necessary to produce good pictures of human beings. Pictures anywhere near the magnificent panorama of passing forms and human characteristics which *Punch* unrolls before us year after year, will be of as much value to future historians as archives full of documents. And only artists of the highest skill can produce them.

The sort of unnatural representation referred to can be set out by any one with some slight natural gift for delineation and learnt by correspondence. It is not caricature, for caricature implies a likeness to original, but simply human semblances badly drawn or rather drawn unnatural according to the individual convention of the drawers. It is a lazy, slovenly misapplication of talent—a prostitution to low use of a gift. It is unfortunate that these pictures appeal to a proportion of the public, and probably create a taste for themselves; just as the mind gets debased by prurient literature and craves for more, so the artistic taste found in some form at least in all human beings gets debased by contemplation of this low form of picture production and comes to like it and to lose taste for higher things. The picture books with over bright and clean boys and girls, birds and beasts, farmyards and country scenes all wished to be beautiful, all led the child to admire the beautiful and to see it in the common things about it. Compare with such those horribly inartistic coloured halfpenny sheets such as may be seen any day being pored over by children of all ages up to adolescence in railway carriages and trams. To use such must surely corrupt taste and spoil the power to appreciate the beautiful. The education of the adult can only be carried on by the adult, but perhaps it would be wise for parents and teachers and all interested in the young to consider this question of the cult of the ugly and to beware of it.

